# The mechanical phenotypic plasticity of melanoma cell: an emerging driver of therapy cross-resistance

**DOI:** 10.1038/s41389-023-00452-8

**Published:** 2023-02-11

**Authors:** Serena Diazzi, Sophie Tartare-Deckert, Marcel Deckert

**Affiliations:** 1 Université Côte d’Azur, INSERM, C3M, Microenvironment, Signaling and Cancer, 06200 Nice, France; 2 Equipe labellisée Ligue Contre le Cancer, Nice, France

**Keywords:** Cancer microenvironment, Melanoma

## Abstract

Advanced cutaneous melanoma is the deadliest form of skin cancer and one of the most aggressive human cancers. Targeted therapies (TT) against BRAF mutated melanoma and immune checkpoints blockade therapies (ICB) have been a breakthrough in the treatment of metastatic melanoma. However, therapy-driven resistance remains a major hurdle in the clinical management of the metastatic disease. Besides shaping the tumor microenvironment, current treatments impact transition states to promote melanoma cell phenotypic plasticity and intratumor heterogeneity, which compromise treatment efficacy and clinical outcomes. In this context, mesenchymal-like dedifferentiated melanoma cells exhibit a remarkable ability to autonomously assemble their own extracellular matrix (ECM) and to biomechanically adapt in response to therapeutic insults, thereby fueling tumor relapse. Here, we review recent studies that highlight mechanical phenotypic plasticity of melanoma cells as a hallmark of adaptive and non-genetic resistance to treatment and emerging driver in cross-resistance to TT and ICB. We also discuss how targeting BRAF-mutant dedifferentiated cells and ECM-based mechanotransduction pathways may overcome melanoma cross-resistance.

## Melanoma cell plasticity: a key component of therapy resistance

Cutaneous melanoma is an aggressive skin cancer with an increasing incidence. Its prognosis is poor in advanced and metastatic stages. Melanoma is a non-epithelial tumor that originates from the malignant transformation of a melanocyte, the cell responsible for pigmentation [1, 2]. The aggressiveness of melanoma is mainly due to a remarkable plasticity of tumor cells creating a significant intra-tumoral heterogeneity associated with resistance to treatment and a high potential for dissemination [3]. Melanoma cell plasticity drives tumor cells diversity along a reversible phenotypic spectrum. This process called phenotype switching involves transcriptional and epigenetic reprogramming ranging from a differentiated melanocytic and proliferative cell state to dedifferentiated mesenchymal-like and neural crest stem-like cell (NCSC) phenotypes with intermediate states [4–8]. The differentiated state displays high levels of the melanocyte lineage-specific transcription factors microphthalmia-associated transcription factor (MITF) and SOX10. Conversely, dedifferentiated melanoma cells are not very proliferative, but particularly invasive, and show low expression of MITF and high expression of mesenchymal [9, 10], invasive [11, 12], extracellular matrix (ECM) [13], and resistance markers such as the receptor tyrosine kinase (RTK) AXL [14]. The dynamic tendency to transition to a mesenchymal phenotype, which can be driven by a variety of tumor microenvironmental stresses including inflammation, nutrient and oxygen deprivation, immune defense or therapies, conveys melanoma cells survival and adaptive capabilities during tumor development and treatment and underlies aggressive traits such as drug resistance and metastatic competence [15–20].

The majority of cutaneous melanomas result from oncogenic driver mutations that lead to constitutive activation of the mitogen-activated protein kinases (MAPK) pathway, with mutations of *BRAF* (B-rapidly accelerated fibrosarcoma, 40-50% of cases), *NRAS* (neuroblastoma ras viral oncogene, 20-30% of cases), or *NF1* (neurofibromatosis type 1, 10-15% of cases) [21]. The discovery that ∼50% of tumors are driven by BRAF^V600^ mutations has led to the development of targeted therapies (TT) based on selective inhibitor of mutant BRAF (vemurafenib, dabrafenib or encorafenib), used clinically in combination with a MEK inhibitor (cobimetinib, trametinib or binimetinib) for the treatment of advanced metastatic melanoma [22–24]. For patients who do not carry the BRAF mutation or who have relapsed after TT, other therapeutic strategies exist consisting in the administration of immune checkpoint blockade (ICB) agents such as anti-PD-1, anti-PDL1, and anti-CTLA4 antibodies aimed at reactivating immune responses against tumor cells [25, 26]. The use of TT and ICB has markedly improved clinical outcome of patients. Yet, despite high response rates to these therapies, the majority of patients do not respond, and many escape these therapies [27].

Several mechanisms of primary, adaptive and acquired resistance to oncogenic BRAF pathway inhibition have been described. Most often, acquired resistance occurs through melanoma genetic evolution leading to reactivation of the MAPK pathway resulting from *de novo* mutations on components of this signaling cascade, including secondary mutations in *NRAS* [28]. Non-genetic mechanisms of drug resistance have obtained increased attention in the last years and their importance for tumor cell adaptation to therapies, drug tolerance and acquired resistance is now recognized [29]. They are linked to tumor cell intrinsic plasticity [30] and in melanoma, they are commonly associated with transcriptional reprogramming and epigenetic changes leading to the activation of alternative survival pathways through upregulation of RTKs such as AXL, PDGFRβ, EGFR or NGFR in a subset of dedifferentiated melanoma cells [3, 7, 14, 17, 31–33]. Upon drug pressure, melanoma cells may adapt to therapy by switching from proliferative to invasive MITF^low^ dedifferentiated melanoma subpopulations, such as neural crest-like and mesenchymal invasive cell states, that impart acquired resistance and tumor relapse [34–36]. Importantly, dedifferentiation and upregulation of genes involved in mesenchymal transition, ECM remodeling and cytoskeletal reorganization has been linked to immune escape and resistance to PD-1 blockade [33, 37–39], revealing possible cross-resistance mechanisms between TT and ICB.

This review summarizes recent evidence underlining the mechanical plasticity of dedifferentiated melanoma cells as a major component of adaptive and non-genetic resistance to TT. We also discuss the potential role of melanoma cell mechanical properties in driving resistance to MAPK-targeted therapies and immunotherapies and how targeting ECM-driven mechanotransduction pathways may be employed to tackle melanoma therapeutic resistance.

## The matrix revolution: tumor microenvironment remodeling in melanoma response to treatment

Tumors are more than cancer cells. Aside genetic alterations intrinsic to tumor cells that drive malignant development, the role of the tumor microenvironment (TME) in cancer progression and therapeutic escape is largely recognized [40–43]. Within the surrounding microenvironment, a dynamic dialog between cancer and stromal cells promotes adaptive resistance to anti-cancer treatment. Among the extrinsic cues that sustain this crosstalk are the release of growth and inflammatory factors, chemical conditions like hypoxia or low nutrients, and the deposition and remodeling of an altered ECM [18, 44–46]. Solid tumors are characterized by a stiffened ECM composed of cross-linked and aligned collagen fibrils. There is increasing evidence that the biophysical properties of tumor-associated ECM promote cell transformation, influence tumor transition states and alter angiogenesis to foster metastasis and compromise treatment efficiency. Hence, ECM dysregulation is viewed as a hallmark of cancer [47]. Tumor-associated fibrosis accompanied with increased ECM deposition and stiffening and unchecked inflammatory signals is now widely accepted as a microenvironmental condition promoting tumor aggressiveness in several types of malignancies, such as breast and pancreatic cancers [48, 49]. Consistently, chronic fibrosis is a well-known risk factor for cancer [50]. Therefore, the effectiveness of anti-cancer treatments requires a thorough understanding of the mechanisms involved in the complex interplay between ECM mechanics, cancer cells and stromal cells. Preventing or reversing pathological ECM remodeling and stiffening or disrupting the cellular response to biomechanical signals is now viewed as a promising approach to enhance response to cancer therapeutics.

Over the past decade, the ECM in the tumor microenvironment has been shown to play a key role in the progression and acquisition of therapeutic resistance in melanoma [51, 52]. Although TT and ICB have improved overall survival in patients with metastatic cutaneous melanoma [27], therapeutic resistance, which involves both tumor cell intrinsic mechanisms and extrinsic cues from the tumor microenvironment, constitutes a major hurdle in the successful treatment of melanoma. Previous studies have revealed that blockade of oncogenic BRAF signaling alters the tumor microenvironment acting both on melanoma-associated fibroblasts (MAF) and melanoma cells to promote therapy escape. First, activation of MAF in the tumor microenvironment exposed to TT triggers the release of soluble pro-survival factors, including hepatocyte growth factor (HGF), from local fibroblasts, which underlie an innate mechanism of drug resistance [42, 43]. The secretion by aged fibroblasts from the melanoma microenvironment of the Wnt-antagonist, secreted frizzled related protein 2 (sFRP2), also impairs tumor response to MAPK-targeted therapies [53]. Another study showed that TT induce complex tumor secretomes in drug-stressed melanoma and human lung adenocarcinoma cells, promoting resistance and tumor progression [54]. Furthermore, upon exposure to TT, autocrine production of TGFβ by melanoma cells can activate local fibroblasts into myofibroblasts to deposit fibronectin that generates a resistance tumor niche, in which melanoma cell adhesion to the fibronectin-enriched ECM amplifies pro-survival signaling activated by HGF released from MAF [55]. The fibronectin-enriched matrix that is produced following the paradoxical stimulation of MAF by BRAF inhibitors also promotes adhesion-dependent signaling through the integrin β1/FAK/SRC axis, which allows melanoma cells to tolerate BRAF inhibition [56]. Importantly, fibrosis also entails inflammatory signals, which have an important role themselves in promoting tumor progression [57] and in shaping a drug-tolerant microenvironment [16, 18, 51, 58]. Together, these studies emphasize the complex interplay between melanoma cells, immune cells, and activated fibroblasts in mediating therapeutic escape.

On the other hand, the critical role of TT-exposed melanoma cells in the pro-fibrotic rewiring of the tumor niche has recently gained increased attention. In fact, the autonomous ability of melanoma cells to produce and shape their own ECM is now described as a major adaptation strategy in response to TT. BRAF inhibition has been shown to increase the production of fibronectin by PTEN-null melanoma cells and adhesion to β1 integrin, which in turn promotes drug resistance through AKT signaling and MCL-1 expression [59]. BRAF inhibition also increases type I collagen synthesis and deposition by melanoma cells in vitro and in vivo, independently of TGFβ signaling [60]. These studies supported the first notion that melanoma residual disease and the resistant niche may be sustained by ECM-derived signals. They also indicated that cooperative remodeling of the cellular microenvironment and the ECM by TT generates a host-tumor niche, which protects melanoma cells from therapeutic insults, paving the way for the development of combination therapies targeting the tumor-derived ECM and the oncogenic BRAF pathway to enhance treatment efficacy.

## Sensing the microenvironment: mechanical forces foster therapy resistance

Therapeutic pressure is a major driver of phenotype plasticity, a pivotal mechanism of non-genetic drug resistance in cancer [29, 30]. In response to MAPK pathway inhibition, some melanoma cells undergo transcriptional reprogramming towards a melanocytic lineage dedifferentiation cell state characterized by the expression of receptor tyrosine kinases such as AXL, PDGFRβ or NGFR [6, 14, 31, 34, 35]. Our recent studies revealed a novel mechanism of adaptation to TT whereby melanoma cells establish a positive mechanosignaling loop powered by autocrine remodeling of a drug protective ECM [13, 51, 61]. This vicious feed-forward mechanical loop confers resistance to TT. While the induction of ECM production by TT on melanoma cells and MAF was previously described, these studies shed light on the role of tumor mechanics in drug adaptation and acquisition of TT-resistance. In vitro, BRAF inhibitor (BRAFi)-resistant melanoma cells characterized by dedifferentiated mesenchymal-like or NCSC-like phenotypes display a pronounced mechanosensitivity and elevated mechanosignaling when plated on rigid collagen substrates [62]. The biomechanical phenotype of these dedifferentiated resistant subpopulations of melanoma cells is supported by an increased nuclear translocation and transcriptional activity of YAP and MRTF [62], two major mechanosensitive transcriptional co-activators [13, 51, 62–65]. Consistent with a prominent role of YAP in therapeutic response, elevated YAP expression is a biomarker of poor response to TT in patients with BRAF-mutant tumors and combined YAP and MAPK inhibition is synthetically lethal in BRAF- and RAS-mutant tumor types including melanoma through synergistic induction of apoptosis [66]. In this context, our studies add a novel biomechanical dimension to previous reports performed with cells growing on tissue culture plastic, which illustrated the tumor cell intrinsic non-ECM-mediated activity of YAP [66, 67] and MRTF [65]. However, it is worth noting that both factors display extrinsic functions, including their role in cancer-associated fibroblasts (CAF) [68, 69], that may also contribute to treatment resistance.

**Fig. 1:**
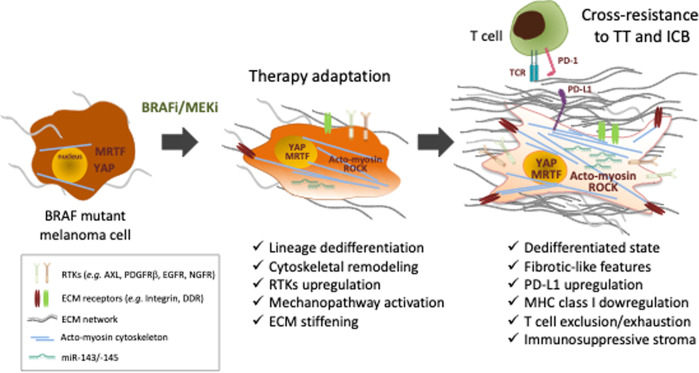
Mechanical phenotypic plasticity in melanoma therapy resistance. Upon treatment with the BRAFi/MEKi combination of targeted therapies (TT), and after an initial response phase, BRAF mutant melanoma cells undergo a series of phenotypic changes during a phase of therapy adaptation, which eventually leads to the acquisition of characteristics favoring cross-resistance to TT and immune checkpoint blockade (ICB) (e.g. anti-PD-1). RTKs receptor tyrosine kinases, ECM extracellular matrix, MHC major histocompatibility complex, TCR T cell antigen receptor.

Further functional analysis showed that poorly differentiated BRAFi-resistant melanoma cells also display a CAF-like phenotype characterized by the upregulation of typical myofibroblast and pro-fibrotic markers, including α-SMA, caveolin 1, MLC2, TAGLN2, FAPα and LOXL2 [13]. Consistently, they display CAF-associated behavior such as generation of actomyosin-dependent mechanical forces and the ability to produce a type I collagen network characterized by aligned fibers, a feature that is typical of activated fibroblasts [13]. This mechanical cell plasticity and myofibroblast/CAF-like activities are also observed during adaptive response to TT (Fig. 1). In contrast, more differentiated BRAFi-resistant melanoma cells, are incompetent to display such CAF-like behavior. Importantly, assembly of an organized ECM is responsible for therapy escape, leading to *de novo* acquisition of resistance. Indeed, treatment-naïve melanoma cells plated on ECM autonomously produced and assembled by dedifferentiated resistant or TT-exposed melanoma cells are protected from the anti-proliferative effect elicited by oncogenic BRAF inhibition [13]. The described ECM-mediated drug protection is mediated by YAP and MRTF activities, as revealed by the observation that depletion of these two transcriptional co-factors prevented drug resistance and implemented TT efficacy. Interestingly, a study showed that RAC1^P29S^, a common mutation in human cutaneous melanoma, drives BRAFi resistance through an SRF/MRTF program, which suppresses melanocytic differentiation, induces a mesenchymal-like phenotype and increases survival and resistance to BRAF inhibitor [70]. An ECM gene expression signature was highly enriched in RAC1^P29S^ tumors compared with RAC1^WT^ tumors [70], suggesting that an MRTF-dependent biomechanical phenotype could also occur in RAC1^P29S^ tumors acquiring resistance to TT.

The biomechanical crosstalk between the tumorigenic ECM and cancer cells may have relevant clinical implications. Indeed, the *de novo* acquisition of a CAF-like phenotype observed in vitro is also demonstrated in vivo using cell-derived xenografts (CDX) and patient-derived xenografts (PDX) models. BRAF inhibition promotes cancer cell-autonomous mechanisms of ECM production and pro-fibrotic features in these xenograft models of melanoma therapeutic responses associated with ECM reprogramming, accumulation of collagen fibers and tumor stiffening in TT-treated mice [13] (Fig. 1). Consistently, disruption of the mechanical crosstalk between the stiff collagen network and melanoma cells by co-administration of TT and the YAP inhibitor verteporfin prevents the fibrotic-like response, enhances TT efficacy and delays the onset of resistance [13]. The notion that the crosstalk between tumor cells and the collagen network is an important element of melanoma resistance to TT is further supported by another study showing upregulated expression of the metalloproteinase and collagenase MT1-MMP in BRAFi-resistant melanoma cells associated with increased β1 integrin/FAK signaling [71]. Targeting this ECM-mediated resistance mechanism using a MT1-MMP inhibitor restored sensitivity to BRAF inhibition in resistant melanoma cells [71]. On the other hand, adhesion of BRAF mutant melanoma cells to ECM generated from MAF confers resistance to TT via the tyrosine kinase receptors for collagens DDR1 and DDR2 and activation of a NIK/NFkB2 survival pathway [72]. In melanoma xenografts, targeting DDR by Imatinib counteracts drug-induced collagen remodeling, induces tumor cell death, delays tumor relapse, and increases survival [72].

Mechanical reprogramming of melanoma cells in response to TT does not only play a role in acquired resistance but it also participates in the early and late adaptation of cancer cells to drug treatment. In line with this notion, we recently uncovered that the mechanical phenotypic plasticity of dedifferentiated melanoma cells exposed to TT relied on the activity of a pro-fibrotic cluster of microRNAs miR-143/-145 [61]. Blockade of the miR-143/-145 cluster prevented the phenotypic transition towards a drug resistant and dedifferentiated invasive cell state. In melanoma cells, we evidenced that the miR-143/-145 cluster targets the actin-bundling protein Fascin 1, which modulates the actin cytoskeleton-ECM crosstalk and mechanopathways through focal adhesion dynamics [61]. In addition, we found that the multi-kinase inhibitor and anti-fibrotic drug nintedanib prevents ECM remodeling and tumor relapse in a syngeneic melanoma model treated with TT and impairs the upregulation of miR-143/-145 in melanoma cells, in part through its inhibitory action on PDGFRβ, which is overexpressed on dedifferentiated melanoma cells. This further supports the concept that targeting the pro-fibrotic rewiring of tumor cells should be considered as a salvage therapy in melanoma [61].

Overall, these studies identify the biomechanical phenotype as a targetable vulnerability of BRAF-mutated melanoma exposed to MAPK therapeutics, and reveal that preventing the pro-fibrotic stromal reaction in response to TT is a viable therapeutic option to overcome non-genetic adaptive drug resistance.

## Targeting mechanopathways to overcome melanoma cross-resistance to targeted therapy and immunotherapy

The pro-fibrotic stroma recently characterized in BRAF-mutant melanoma treated with TT is the hallmark of other solid malignancies, and in addition to promote tumor initiation, metastatic dissemination and drug resistance, it has been described to be responsible for immune evasion [50, 73]. A general example is the fibrotic state of desmoplastic tumors, which leads to immunosuppression through several mechanisms [74]. The notion that aberrant organization or stiffening of the ECM may impede T cell migration and infiltration in the tumor has also recently emerged. FAK signaling in pancreatic ductal adenocarcinoma is associated with desmoplasia and impaired cytotoxic T lymphocytes infiltration [75], while the CXCR4/CXCL12 axis triggers a similar effect in breast metastasis [76]. Moreover, several studies point out that several features of fibrotic tumors, such as hypoxia and the presence of tumor-associated macrophages, impede T cell infiltration [77, 78]. Consequently, normalization of the tumorigenic ECM has been shown to reverse immune exclusion and improve ICB outcomes in preclinical mouse cancer models. One of the most efficient anti-fibrotic strategies so far developed to implement the efficacy of immune checkpoint inhibitors is targeting TGFβ signaling [79, 80]. However, the pleiotropic effects of TGFβ make it challenging to exploit this therapeutic option in the clinic. A recent study deepens the notion of tumor-associated fibrosis as a negative regulator of anti-tumor immunity and ICB response [81]. Using several preclinical mouse tumor models with heterogeneous stroma, the authors show that ECM remodeling and stiffening alter intra-tumoral T cell migration. Conversely, normalization of tumorigenic ECM and collagen crosslinking through the inhibition of lysyl oxidase (LOX) reduced ECM deposition and stiffness, which enhanced T cell migration and increased the efficacy of anti-PD-1 therapy [81]. Recent observations in the non-BRAF-mutated B16-F10 melanoma model indicate that inhibition of the DDR2 collagen receptor [82] or CAF-mediated fibrosis by nintedanib [83] improved the antitumor activity of ICB, reinforcing the idea that targeting ECM remodeling is a promising therapeutic approach to enhance immunotherapies in cancer.

The contribution of the fibrotic-like phenotype and biomechanical plasticity to immune evasion has critical clinical implications for other cancers currently treated with ICB, including melanoma (Fig. 1). Indeed, in TT-treated patients with BRAF-mutant melanoma, it has been shown that the acquisition of a dedifferentiated cell state characterized by the expression of mesenchymal genes, and genes involved in cell adhesion and migration, ECM remodeling and wound healing is not only typical of resistance to TT but also of ICB resistance [6, 33, 39, 84]. In melanoma patients, the dedifferentiated subpopulation with high expression of the NCSC-associated receptor NGFR is associated with immune exclusion and resistance to anti-PD-1 therapy [85]. Remarkably, the NCSC-like cellular state in BRAF-mutated melanoma has also been associated with the development of non-genetic resistance to MAPK-targeted therapies in a NGFR/FAK/AKT-dependent manner [35, 36]. Conversely, resistance to PD-1 blockade has been linked to TGFβ signaling, which drives a treatment resistant dedifferentiated cell state and transcriptional downregulation of MHC class I in melanoma [86]. In this context, it is interesting to note that lineage dedifferentiation of melanoma cells that is induced by the proinflammatory cytokine TNFα also causes the resistance to T-cell adoptive cell transfer therapies [58, 87]. On the other hand, a T cell exhaustion phenotype associated with YAP signature enrichment in the tumor cell compartment is a typical feature of acquired TT resistance [33]. Therefore, the presence of subpopulations of dedifferentiated and mechanically plastic melanoma cells may predict cross-resistance to TT and ICB. However, a recent study showed that, in a syngeneic mouse model, cross-resistance to ICB can also be driven in TT-resistant melanoma by reactivation of the MAPK pathway, which promotes an immunosuppressive microenvironment with dysfunctional dendritic cells [88]. This study, and others [35, 84, 89, 90] illustrate the complexity of acquired resistance mechanisms to anti-cancer therapeutics. Nevertheless, consistent with the concept of cross-resistance associated with the mechanophenotype of melanoma cells, another study identified a key role for cytoskeletal remodeling and the ROCK-actomyosin mechanosensing pathway in resistance to both TT and ICB [37]. Targeting ROCK or myosin II causes death of TT and ICB-resistant melanoma cells via lethal ROS induction and unresolved DNA damage. In addition to these intrinsic actions, ROCK-myosin II blockade limits the action of immunosuppressive myeloid and lymphoid cells, thereby improving oncogenic BRAF oncogenic pathway inhibition and anti-PD-1 efficacy [37]. Cytoskeletal plasticity should therefore be considered an intrinsic vulnerability of therapy resistant melanoma cells that can be exploited in the clinic.

## Perspectives

Therapeutic resistance remains a major challenge in the clinical management of metastatic melanoma. Besides shaping the tumor microenvironment, cancer therapies impact tumor cell plasticity and phenotypic diversity to promote drug tolerance and compromise treatment efficacy [29, 30]. Recent studies have highlighted melanoma cell phenotypic plasticity as a major component of adaptive and non-genetic drug resistance, a phenomenon that precedes irreversible genetic resistance [29]. Furthermore, unlocking phenotypic plasticity and non-mutational epigenetic reprogramming has been recently added to the hallmarks of cancer [73]. In this context, the autonomous ability of mechanosensitive dedifferentiated CAF-like melanoma cells to produce and remodel their own ECM is now emerging as a major mean to evade MAPK-targeting therapies and immunotherapies and a source of cross-resistance. Yet, the mechanism of biomechanical adaptation of cancer cells to treatment, which fuels tumor relapse, also reveals new vulnerabilities. Targeting the mechanical phenotype displayed by aggressive dedifferentiated mesenchymal-like BRAF-mutant melanoma cells and the fibrotic-like features of melanoma tumor microenvironment in combination with current TT and ICB clearly opens promising therapeutic avenues. For example, repurposing compounds used in the treatment of fibrotic diseases represents an attractive approach to target the biomechanical adaptation of melanoma to treatment [51]. This can be achieved by normalizing therapy-induced ECM stiffening using therapeutics against TGFβ signaling [91], targeting LOX and LOXL2 collagen cross-linkers [92] or using approved anti-fibrotic drugs such as nintedanib [93], which has shown promising activity in a preclinical model of BRAF mutant melanoma exposed to TT [61]. Blocking ECM signaling by targeting integrins and their associated FAK kinase [36, 75, 94], or DDR1/2 collagen receptors [72, 82, 95], also has broad potential to improve anti-cancer therapies. Finally, blocking the aberrant intracellular mechanotransduction pathways that are promoted by ECM assembly and stiffening, including RHO GTPase-mediated actomyosin cell contractility [37] and transcriptional activity by the mechanosensors YAP [13, 66, 96, 97] and MRTF [65, 70, 98], represents an alternative approach to overcome therapeutic resistance. Targeting pathological and stromal cells mechanics emerges as a new field of medical sciences (the so-called mechanomedecine) that holds great potential to limit the progression of melanoma and other cancers with major implications in the clinic (for detailed reviews of the current mechanobiology-directed trials the reader should refer to [46, 91]).

The coming years should see whether the implementation of therapeutics targeting the mechanical dialog between cancer cells and their microenvironment will help to overcome cross-resistance and finally benefit patients relapsing on targeted and immune therapies.

## Data Availability

All data presented in the current study are publicly available in the MEDLINE database in accordance with the reference list.
